# Development of maize plant dataset for intelligent recognition and weed control

**DOI:** 10.1016/j.dib.2023.109030

**Published:** 2023-03-01

**Authors:** Olayemi Mikail Olaniyi, Muhammadu Tajudeen Salaudeen, Emmanuel Daniya, Ibrahim Mohammed Abdullahi, Taliha Abiodun Folorunso, Jibril Abdullahi Bala, Bello Kontagora Nuhu, Adeyinka Peace Adedigba, Blessing Israel Oluwole, Abdullah Oreoluwa Bankole, Odunayo Moses Macarthy

**Affiliations:** a Department of Computer Engineering, Federal University of Technology, P. M. B. 65, Minna, Niger State, Nigeria; b Department of Crop Production, Federal University of Technology, P. M. B. 65, Minna, Niger State, Nigeria; c Department of Mechatronics Engineering, Federal University of Technology, P. M. B. 65, Minna, Niger State, Nigeria

**Keywords:** Maize images, Precision agriculture, Autonomous robot, Herbicides

## Abstract

This paper focuses on the development of maize plant datasets for the purposes of recognizing maize plants and weed species, as well as the precise automated application of herbicides to the weeds. The dataset includes 36,374 images captured with a high-resolution digital camera during the weed survey and 500 images annotated with the Labelmg suite. Images of the eighteen farmland locations in North Central Nigeria, containing the maize plants and their associated weeds were captured using a high-resolution camera in each location. This dataset will serve as a benchmark for computer vision and machine learning tasks in the intelligent maize and weed recognition research.

**Specifications Table**

**Table:** 

Subject	Engineering
Specific subject area	Computing Vision applied to annotated maize and weed images critical for development of computer-based autonomous systems that reduce human involvement in crop production activities, especially maize recognition, weed identification and control in a maize farmland.
Type of data	Image
How the data were acquired	The raw wet and dry seasons images have been captured using a high-resolution digital camera during the weed survey. Specifically, the 24.3 MP digital image camera (Sony A6000,) was used to acquire the raw image of the maize as well as the weeds in situ on various farmlands within the study area. The images, as well as video frames, were acquired at a distance of one foot from the crop while maintaining the camera at a uniform height. The annotation of the annotated image was done using the Labelmg suite.
Data format	Raw Analyzed Filtered
Description of data collection	The data were acquired from the survey of the various maize-based farmlands during the study. They were recorded in both video and image formats, with video clips converted into image frames. The raw data were then filtered, annotated, and saved.
Data source location	• Federal University of Technology, Minna • Niger State/Bosso, Gbako, Katcha and Lapai Local Government Areas • Nigeria • The coordinates of the various fields visited were obtained as primary data during field visits (survey) and used as the explanatory variables in Table 2.
Data accessibility	Repository name: Mendeley Data. Data identification number: DOI: 10.17632/jjbfcckrsp.2 Direct URL to data: https://data.mendeley.com/datasets/jjbfcckrsp
Related research article	M.T. Salaudeen, E. Daniya, O. M. Olaniyi, T. A. Folorunso, J. A. Bala, I. M. Abdullahi, B. K. Nuhu, A. P. Adedigba, B. I. Oluwole, O. A. Bankole and O. M. Macarthy, Phytosociological survey of weeds in irrigated maize fields in a Southern Guinea Savanna of Nigeria. Front. Agron. (2022) 4:985067. https://doi.org/10.3389/fagro.2022.985067

## Value of the Data

This data has immense value to the scientific community. The following points highlights some of the reasons why this data is important to the scientific community:

• The published weed database will provide baseline information to researchers working on weed recognition and control, using computer vision and machine learning for intelligent herbicide application in maize for increased crop yield.
• The dataset generated will be relevant to weed scientists, agronomists, botanists and farmers interested in precision and smart agriculture, involving automated weed recognition and control in maize farmland.
• Government agencies, policymakers and agribusiness will also be guided on policies and programs that will drive the development, adoption and regulation of technologies and products that are likely to be developed with the availability of this data.
• The dataset can also be used for weed species and maize plant recognition by annotating the area of interest, extracting relevant features and training a machine/deep learning model for recognition.
• The trained model from the dataset can be used to develop autonomous systems for herbicide spraying and other related applications.

## 1 Objective

The dataset was acquired locally in Niger State, North Central Nigeria, covering four local government areas. The goal is to localize the dataset in order to meet the urgent demands of the local farmers. Due to the varying types of soil and land in those places, the authors found that different agricultural practices were implemented in the various local government districts that were visited. In addition, review of literatures in [3], [4], [5], [6], [7], [8], [9], [10], indicated that data of this nature were not widely available; therefore, the authors had to curate a dataset for the public and save it in a public domain for use.

## 2 Data Description

The dataset in [2], contains images of maize plants and weed species. The dataset contains 36,874 images in total and is stored in four folders: Annotated Maize-Weed Images, Data Description and Questionnaire, Dry Season Maize Weed Images, and Wet Season Maize Weed Images. The Dry Season contains 18,187 images captured during the dry season farm survey. The Wet Season contains 18,187 images captured during the wet season farm survey, and the Annotated contains 500 annotated images selected from the Dry Season survey saved in JSON, XML, and txt format. The images of the raw wet and dry seasons were captured using a high-resolution digital camera during the Maize-Weed survey carried out on 18 farm locations in the North Central part of Nigeria. In contrast, the annotation of the images was achieved using the Labelmg suite, an open-source annotation tool. The summary of the data is presented in Table 1.

**Table 1:** Dataset description.

Data	Description
Dry Season Maize Weed Image	This folder contains 18,187 raw images of Maize-Weed acquired during the Dry farming Season (acquired in April 2022).
Wet Season Maize Weed Image	This folder contains 18,187 raw images of Maize-Weed acquired during the Wet farming Season (acquired in August 2022) and 18,187 images.
Annotated Maize-Weed Image	This folder contains 500 annotated images of Maize-Weed acquired during the Dry Season (acquired in April 2022). The annotation was done using the Labelmg tool.
Data Description and Questionnaire	This folder contains the description of data and the questionnaire administered to farms during the survey in April and August 2022.

## 3 Experimental Design, Materials and Methods

Several farms in four (Bosso, Gbako, Katcha, and Lapai) different Local Government Areas (LGAs) in Niger State, Nigeria were visited. The details of the farms visited and their geographic information are shown in Table 2. A total of 18 farms were visited with the lowest elevation of 76 metres above sea level(masl) and the highest elevation of 225 metres above sea level (masl). The longitude and latitude are also shown in the Table 2. Primary data were collected from the respondents during the field visits (survey) with use of structured questionnaire.

**Table 2:** Details of farms visited.

Local Government Area	Location	Latitude (^0^N)	Longitude (^0^E)	Altitude (m)
Bosso	Anguwan-Shaba_01	9.53589	6.58369	225
Bosso	Anguwan-Shaba_02	9.53562	6.58379	214
Gbako	Sabon-Gida_01	9.24997	6.16158	97
Gbako	Sabon-Gida_02	9.24835	6.16103	95
Gbako	Sabon-Gida_03	9.24729	6.16051	94
Gbako	Sabon-Gida_04	9.24357	6.1626	76
Gbako	Sabon-Gida_05	9.2445	6.16134	93
Gbako	Sabon-Gida_06	9.25229	6.16061	90
Gbako	Sabon-Gida_07	9.25239	6.15988	96
Katcha	Katcha_01	9.25146	6.16168	99
Katcha	Katcha_02	9.25192	6.16192	100
Katcha	Katcha_03	9.25264	6.16187	96
Katcha	Katcha_04	9.25264	6.16187	158
Katcha	Katcha_05	9.25212	6.15733	95
Katcha	Katcha_06	9.24982	6.16652	85
Katcha	Katcha_07	9.25062	6.16691	79
Lapai	Lapai GGSS-Day_01	9.02059	6.57805	140
Lapai	Lapai GGSS-Day_02	9.01243	6.58276	146

**Fig. 1:**
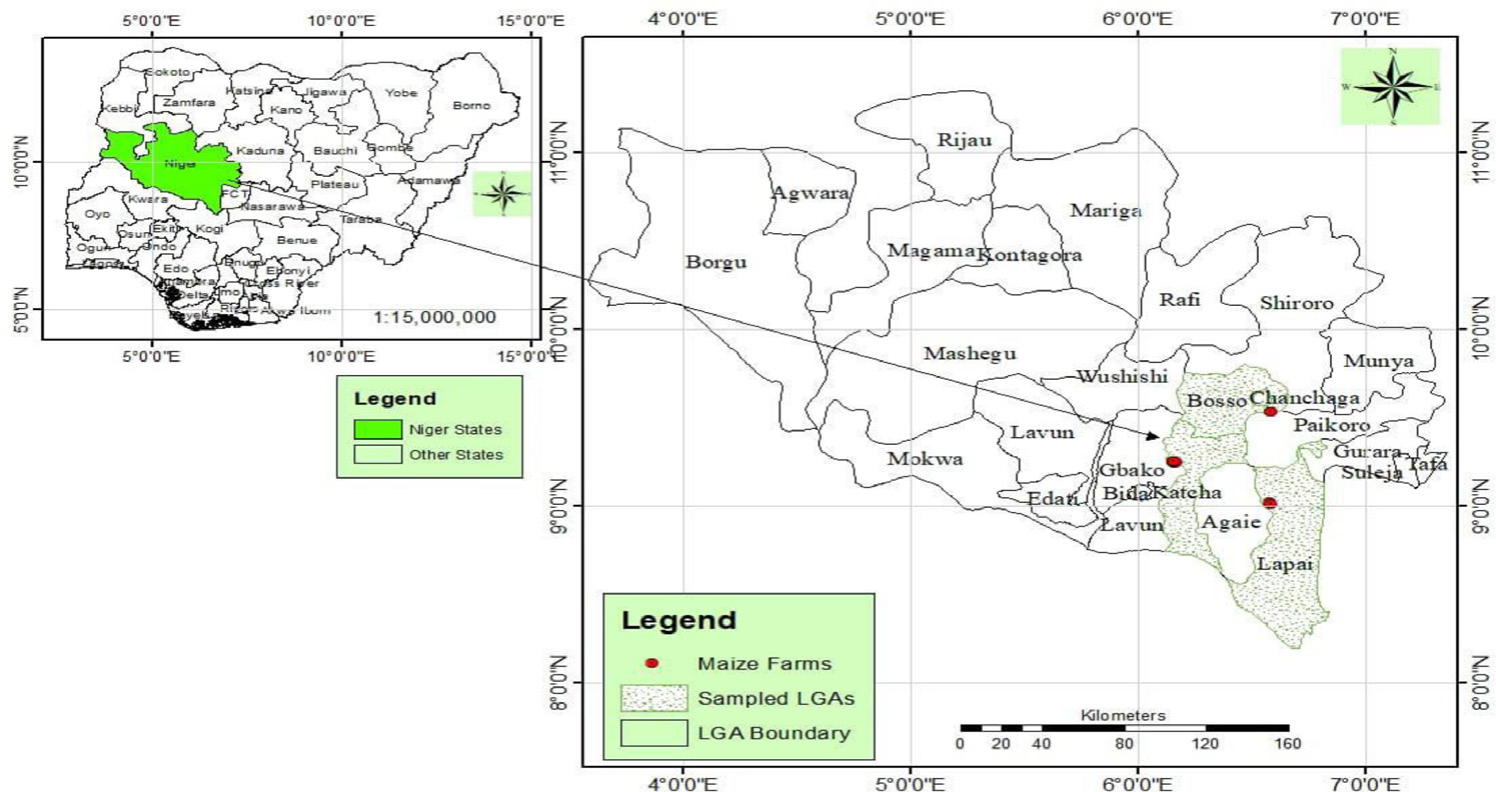
Map of Nigeria showing the Local Government Areas of Niger State surveyed for weeds data capturing [1].

Maize-based cropping system farms were surveyed in Bosso, Gbako, Katcha and Lapai LGAs (Fig. 1) between February and March 2022. A handheld Global Positioning System machine (GPS- 4300; Ethrex 10 Garmin, Taiwan) was used to record the coordinates of each location, and the data were used to map the surveyed locations [1,11].

The dataset contains images of maize crops and their accompanying weeds that alongside farm. These images were taken on field trips to 18 different maize farmlands within four (4) Local Government Areas of Niger State, Nigeria, West Africa. The data were collected from irrigated maize fields under traditional maize cultivation practices. Raw data of maize and weeds were captured in situ using a 24.3 MP digital picture camera (Sony A6000). The images and video frames were taken one foot away from the crop, with the camera held at a constant height. The images were acquired in a 'handheld' manner. This implies that the photographer moved through the farmland while manually holding the camera. This manual data acquisition process was required because the terrain of the farmland and the spacing of the ridges did not allow for the use of a vehicle in which the camera could be mounted. The images were checked individually to ensure they were in focus and the blurry images removed from the dataset. Direct interviews and open discussion with the maize farmers were carried out on the status, perception of weed infestation and methods of weed control, and the need for a technology that can detect weeds and apply herbicide in their fields. This information was obtained from the maize farmers using a guided questionnaire during the survey.

After data acquisition, the data were filtered to remove noise and outliers from the images. The filtered images were stored in the ‘JPEG’ format, the same as the acquired images to preserve data integrity. Furthermore, the data was annotated with the open-source annotation tool 'LabelImg' and saved as XML and JSON formats.

## Ethics Statements

The authors collected data from various farms in the study area. The farm owners were contacted, and permission was obtained from them by the research team. In addition, the surveys were conducted in the presence of the farm owners, and they were interviewed accordingly. Sample of the administered questionnaire is available in the data repository at https://data.mendeley.com/datasets/jjbfcckrsp.

## CRediT authorship contribution statement

**Olayemi Mikail Olaniyi:** Conceptualization, Resources, Writing – original draft, Writing – review & editing, Supervision, Project administration, Funding acquisition. **Muhammadu Tajudeen Salaudeen:** Conceptualization, Investigation, Resources, Writing – review & editing, Supervision, Project administration, Funding acquisition. **Emmanuel Daniya:** Conceptualization, Methodology, Validation, Investigation, Writing – review & editing, Funding acquisition. **Ibrahim Mohammed Abdullahi:** Methodology, Writing – original draft, Writing – review & editing. **Taliha Abiodun Folorunso:** Conceptualization, Data curation, Writing – original draft, Funding acquisition. **Jibril Abdullahi Bala:** Conceptualization, Writing – original draft, Funding acquisition. **Bello Kontagora Nuhu:** Methodology, Software, Formal analysis. **Adeyinka Peace Adedigba:** Methodology, Software, Formal analysis. **Blessing Israel Oluwole:** Methodology, Validation, Investigation. **Abdullah Oreoluwa Bankole:** Methodology, Software, Data curation. **Odunayo Moses Macarthy:** Methodology, Software, Formal analysis, Data curation.

## Declaration of Competing Interest

The authors declare that they have no known competing financial interests or personal relationships that could have appeared to influence the work reported in this paper.

## Data Availability

Maize-Weed Image Dataset (Original data) (Mendeley Data).
